# Small for gestational age and age at menarche in a contemporary population-based U.S. sample

**DOI:** 10.1371/journal.pone.0309363

**Published:** 2024-09-06

**Authors:** Sruchika Sabu, Hope Corman, Kelly Noonan, Nancy E. Reichman, Kirsten B. Kuhn, Sally Radovick

**Affiliations:** 1 Department of Pediatrics, Robert Wood Johnson Medical School, New Brunswick, New Jersey, United States of America; 2 Department of Economics, Rider University and National Bureau of Economic Research, Lawrenceville, New Jersey, United States of America; 3 Department of Economics, Princeton University, Princeton, New Jersey, United States of America; 4 Child Health Institute of New Jersey, Rutgers University, New Brunswick, New Jersey, United States of America; 5 School of Public and International Affairs, Princeton University, Princeton, New Jersey, United States of America; Universitair Kinderziekenhuis Koningin Fabiola: Hopital Universitaire des Enfants Reine Fabiola, BELGIUM

## Abstract

Children born small for gestational age (SGA) may be at risk for earlier puberty and adverse long-term health sequelae. This study investigates associations between SGA and age at menarche using secondary data on 1,027 female children in a population-based U.S. birth cohort that over-sampled non-marital births, which in the U.S. is a policy-relevant population. SGA was defined as <10^th^ percentile of weight for gestational age compared to the national U.S. distribution. We estimated unadjusted and adjusted Ordinary Least Squares (OLS) models of associations between SGA and age at menarche in years, as well as unadjusted and adjusted logistic regression models of associations between SGA and early menarche (before age 11). SGA was not significantly associated with earlier age at menarche, even when adjusting for maternal sociodemographic characteristics, prenatal smoking, and maternal pre-pregnancy overweight and obesity. Similarly, SGA was not significantly associated with the odds of menarche occurring before age 11. However, maternal non-Hispanic Black race-ethnicity, Hispanic ethnicity, and pre-pregnancy obesity all had independent associations with average earlier age at menarche and menarche before age 11. Thus, maternal risk factors appear to play more influential roles in determining pubertal development.

## Introduction

Menarche is a significant developmental milestone experienced by girls, and its timing influences biological, social, and psychological development. Over the last century, many developed countries saw a rapid decrease in the average age of menarche [1]. In some countries, the decline has recently stabilized. For example, the National Center for Health Statistics reports that the average age at menarche in the United States has been about 12.5 years since 1995 [2]. However, the prevalence of early menarche continues to increase in other countries; e.g., a 2020 study found that the prevalence of early menarche (defined as occurring before 10.5 years of age in this study) increased from 1.8% in 2006 to 3.2% in 2015 in Korea [3].

Early menarche is usually defined using standard deviation, percentile, or age cutoffs, and has been variably defined as menarche at a time before the age of 10 through 12 years. Early menarche should be distinguished from *premature* menarche, which is defined as the onset of vaginal bleeding in girls less than 7 years of age and suggests a pathologic process [4]. Early menarche is a concerning phenomenon due to its significant ramifications for the timing of menopause [5] and its relation to obesity [6]. Further, girls with early menarche are more likely to experience depressive symptoms and engage in health risk behaviors that adversely impact health in adulthood [7], although it has not been established whether those associations are confounded by socioeconomic factors. Early menarche has also been associated with an increased risk of developing cardiovascular disease [8], asthma [9], polycystic ovarian syndrome [10], and endometriosis [11]. Because girls who experience early menarche are vulnerable to adverse outcomes, the factors that influence the age at menarche warrant investigation.

Studies have shown that being born small for gestational age (SGA) can result in long-term adverse health sequelae, including early puberty and subsequent short stature [12], obesity [13], and metabolic disorders in adulthood [14]. SGA is a heterogeneous condition that may result from diverse factors, including genetic abnormalities, maternal medical conditions, substance use, demographics, diet, pregnancy course, and past obstetric history [15]. One of the proposed mechanisms relating SGA to early menarche is a phenomenon known as “catch-up growth,” which occurs early in childhood and is associated with increased central adiposity, premature pubarche, earlier puberty, and earlier menarche [12, 13, 16].

Only a few prospective cohort studies have investigated linkages between SGA and age at menarche. In a cohort of children that were followed into adolescence in Sweden (born 1973–1977), girls that were SGA had menarche an average of 5 months earlier than girls born appropriate for gestational age (AGA) [17]. Another study in Korea that followed 30 SGA subjects (born 1988–1999) found that age at menarche did not differ from the population average; however, AGA subjects were not used as controls [18]. In the Danish Puberty Cohort (born 2000–2003), the age at menarche in girls born SGA was about 2 months earlier than that of girls born AGA [19]. In a cohort in Hong Kong (born 1997), no correlation was observed between SGA and the timing of puberty using Tanner staging rather than menarche to define puberty [20]. Recently, a Polish study found that SGA girls with catch-up growth had significantly earlier menarche than girls born AGA or SGA without catch-up growth [21]. The findings from these studies in relatively homogenous populations may not generalize to the U.S., which has a unique racial-ethnic composition. Our study adds to this body of knowledge by investigating linkages between SGA and age at menarche using data from a national U.S. birth cohort study that oversampled non-marital births, which in the U.S. are strongly associated with racial-ethnic minority status [22] and poverty [23] and accounted for 39.8 percent of U.S. births in 2022 (the latest year of data available) [24].

## Materials and methods

### Data

We used data from the Future of Families and Child Wellbeing (FFCWB) study, a population-based national birth cohort study of 4,898 births in large U.S. cities from 1998 to 2000 that conducted postpartum (baseline) interviews with the mothers and fathers and follow-up interviews with the parents and children at subsequent time points. Additional information was collected from the mothers’ and children’s medical records from the birth hospitalization. The availability of medical records depended primarily on the administrative processes of hospitals rather than the decisions of respondents to make their records available. Additional information was taken from interviews with the children that were conducted when they were 15 years old. We focused on children who were assigned as female at birth.

The FFCWB cohort was population-based because it used a probability sample of cities, hospitals, and births within hospitals. Non-marital births were oversampled by design; that is, marital births were no longer included when hospital-specific quotas were reached. The reason for the oversampling was that unmarried parents and their children had been underrepresented in national U.S. datasets. About three-fourths of the mothers in the study were unmarried when they gave birth [25, 26]. Because non-marital childbearing is strongly associated with poverty in the U.S., as is racial/ethnic minority status, the FFCWB sample had high fractions of poor, Black, and Hispanic parents.

The analyses were performed using de-identified third-party data that are available to the public through a restricted data use contact from the Future of Families & Child Wellbeing Study at Princeton University [27], which obtained informed consent from all participants per all relevant guidelines and regulations [26]. Data was accessed to conduct this study on August 15, 2022. The authors had no access to information identifying individual participants for this study, which did not involve data collection.

### Measures

#### Small for gestational age (SGA)

SGA was defined as <10^th^ percentile of sex-specific birth weight for gestational age. Gestational age was calculated as the number of completed weeks between the date of the first day of the mother’s last menstrual period (LMP) and the child’s birthday. The respondent’s birth weight for gestational age was compared to the national U.S. sex-specific reference distribution of LMP-based SGA from 1999–2000 [28] when most of the FFCWB sample members were born.

#### Age at menarche

Age at menarche was measured in months and was reported in the 15-year survey, in which girls were asked whether they had ever had a menstrual period and, if so, to report their age in years and months (e.g., 13 years and 2 months old) when menarche occurred. These questions were supplemented with information from girls’ diary data at age 15 and mothers’ reports about the daughters’ menarche during a 9-year survey. The information on the timing of menarche came from the 15-year survey for approximately 97 percent of cases. For approximately one-quarter of girls, we know their age at menarche in years but not the exact month. In those cases, we set the months equal to 6 and created a binary variable for missing data on months. In supplementary analyses, we dropped the cases with missing data on months. Age at menarche was also evaluated as a binary variable for whether the respondent had experienced menarche before age 11, a cut-off used in previous studies [29].

#### Other analysis variables

The analyses included maternal sociodemographic characteristics, which the mother reported in her postpartum survey. They included her age, race-ethnicity, foreign-born status, education, marital status, parity (first birth vs. higher-order birth), and whether the delivery was insured by Medicaid (a government-funded health insurance program available to low-income pregnant women in the U.S.) (Table 1). Some analyses additionally included pre-pregnancy obesity, pre-pregnancy overweight, and prenatal cigarette smoking. Pre-pregnancy obesity and overweight were based on the height and pre-pregnancy weight recorded in the mother’s medical record, which was used to compute body mass index (weight in kilograms divided by height in meters squared) and classify mothers as overweight or obese using established ranges (25.0 to <30 indicating overweight; 30 or higher indicating obese) [30]. The 14% of mothers with missing data on pre-pregnancy weight were coded as having been normal or underweight. We also controlled for a binary indicator for missing data on pre-pregnancy weight. We assessed sensitivity to excluding the cases with missing pre-pregnancy weight data. For prenatal smoking, we coded the mother as ever having smoked cigarettes during pregnancy if there was an indication of such in the medical records or the mother indicated a positive number in response to a question in the postpartum interview about how many cigarettes she smoked per day during the pregnancy. Indicators were included in the models of SGA in months for missing months (i.e., the respondent reported her age in whole years when menarche began).

**Table 1:** Outcomes and sample characteristics, overall and by SGA.

	Full Sample	Not SGA	SGA	P-value
**Timing of menarche**				
Age, mean months (s.d.)	147.5 (15.8)	147.7 (15.6)	146.4 (17.3)	0.41
Before age 11	0.14	0.14	0.19	0.15
**Maternal sociodemographic characteristics**				
Race-ethnicity				
Non-Hispanic White	0.21	0.21	0.19	0.72
Non-Hispanic Black	0.50	0.48	0.60	**0.01**
Hispanic	0.27	0.28	0.19	**0.04**
Other race	0.03	0.03	0.02	0.35
Education				
High school graduate	0.67	0.68	0.62	0.16
Less than high school	0.33	0.32	0.38	0.16
Nativity				
US born	0.86	0.85	0.92	0.06
Foreign born	0.14	0.15	0.08	0.06
Age, mean years				
< 20	0.17	0.17	0.23	0.10
20–34	0.74	0.75	0.68	0.10
35+	0.09	0.08	0.09	0.76
Relationship with child’s father				
Married	0.24	0.26	0.14	**0.00**
Cohabiting but not married	0.38	0.38	0.37	0.94
Neither married nor cohabiting	0.38	0.37	0.49	**0.01**
Parity				
First birth	0.39	0.38	0.45	0.15
Higher-order birth	0.61	0.62	0.55	0.15
Health insurance for birth				
Medicaid	0.64	0.63	0.76	**0.00**
Other	0.36	0.37	0.24	**0.00**
**Other maternal variables**				
Pre-pregnancy weight				
Obese	0.24	0.25	0.19	0.22
Overweight (but not obese)	0.18	0.19	0.18	0.85
Normal or underweight	0.58	0.57	0.63	0.23
Prenatal smoking				
Any	0.2300	0.21	0.44	**<0.01**
None	0.77	0.79	0.56	**<0.01**
N	1,003	885	118	

Notes: SGA = small-for-gestational age = <10th percentile of sex-specific birth weight for gestational age; s.d. = standard deviation. SGA was based on birth weight and LMP-based gestational age and compared to the national U.S. sex-specific distribution of LMP-based SGA from 1999–2000. Figures are column proportions unless indicated otherwise. All maternal characteristics were assessed from the postpartum interviews. Pre-pregnancy obesity, pre-pregnancy overweight, and smoking were assessed from information in the mother’s medical charts from the birth hospitalization. Calculations are based on sample used for analyses of age at menarche in months. P-values are for differences by SGA at 5% level using a two-tailed t-test (statistically significant p-values in bold).

### Study sample

**Fig 1:**
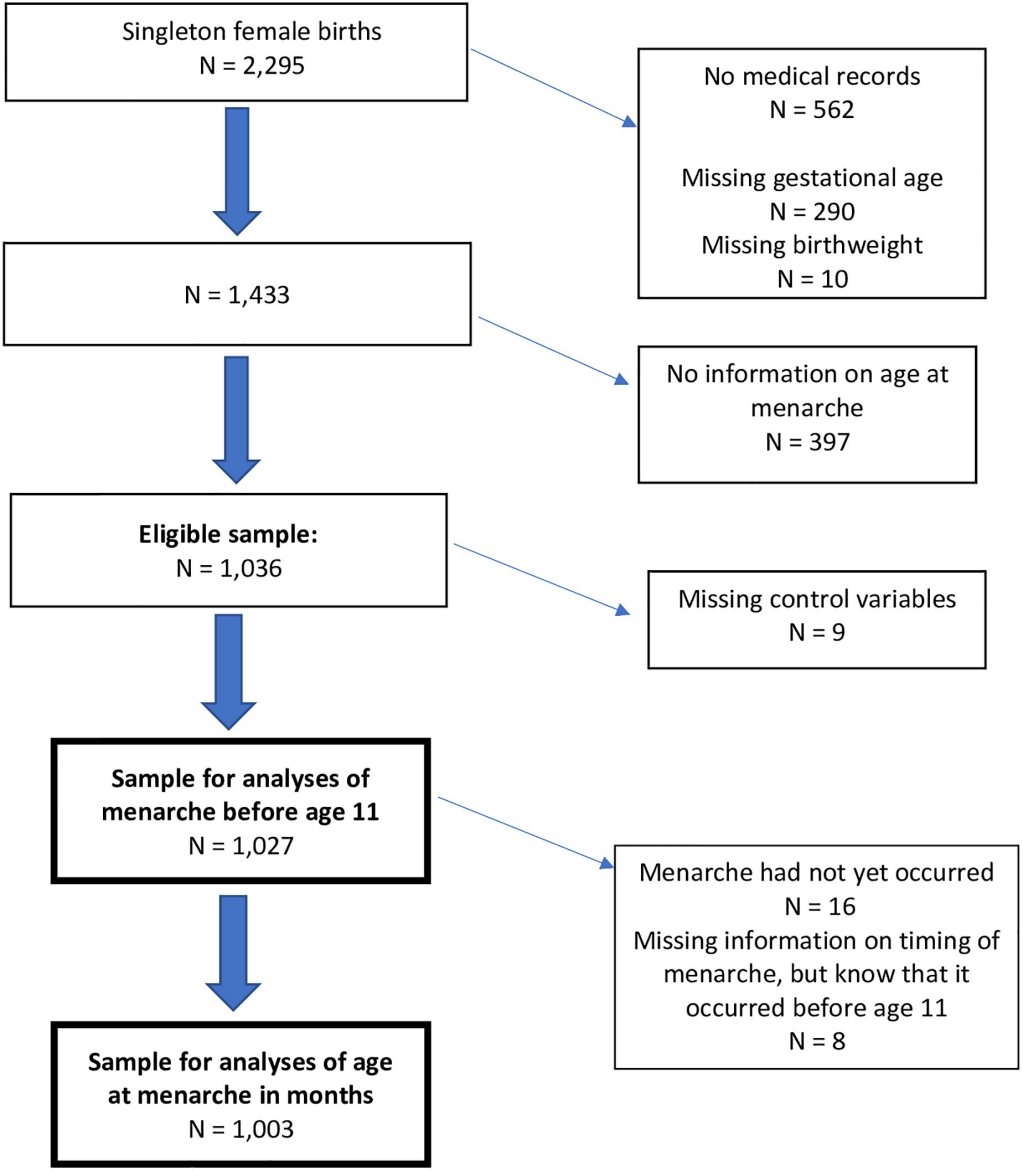
Derivation of analysis sample.

Of the 2,295 female singleton newborns in the FFCWB study, 562 had no available medical record data, and another 300 had missing data on birth weight or the date of the mother’s last menstrual period, both of which were used to compute small for gestational age. Of the 1,433 remaining cases, 387 had no information on the timing of menarche. This resulted in an eligible sample of 1,036 cases (Fig 1). Of those, 9 had incomplete data on other analysis variables, resulting in a sample of 1,027 cases for analyses of menarche before age 11. From the 1,027 cases, 16 who had not yet experienced menarche and 8 who had missing information on the timing of menarche (but it was known that they had menarche prior to age 11) were excluded from analyses of menarche in months, resulting in a sample of 1,003 for that outcome.

### Statistical analysis

First, we compared our analysis sample of 1,003 to the cases in the full cohort of 2,295 that were not in that sample using two-tailed t-tests. Second, we documented age at menarche and maternal characteristics in the analysis sample, both overall and by SGA status. Third, we estimated unadjusted and adjusted linear Ordinary Least Squares (OLS) models of associations between SGA and age at menarche in months. Fourth, we estimated unadjusted and adjusted logistic regression models of associations between SGA and menarche before age 11.

Finally, we estimated sets of supplementary models that alternatively used a different cutoff for SGA (the 25% percentile of birth weight for gestational age) that a recent study found was associated with developmental concerns [31], added a control for the number of completed weeks of gestation, restricted the sample to full term births (37–41 weeks), restricted the sample to children that weighed > 2500 grams at birth, restricted the sample for both outcomes to girls who reported month of menarche as well as the year, used the smaller sample of 1,003 in the logistic regression models, restricted the sample to cases with non-missing pre-pregnancy weight, and used inverse probability weights to account for sample loss from the initial sample of 2,295 cases. We also estimated models that interacted SGA with weight gain between birth and 1 year to assess potential differential associations between SGA and age at menarche by a measure of postnatal catch-up growth that was available in the data.

In all sets of models, the first adjusted model included maternal sociodemographic characteristics, and the second further included maternal pre-pregnancy obesity, overweight, and prenatal smoking. All estimates are presented with 95% confidence intervals.

Analyses were conducted using Stata Version 18 (Stata Corp, College Station, TX). This study was approved by the Institutional Review Boards of Rutgers and Princeton Universities. All data analysis was conducted at Princeton University.

## Results

The mothers of children in the analysis sample did not differ significantly from those who were not in the sample based on race, ethnicity, marital status, age, first birth, and Medicaid coverage for the birth, but they were less likely to be foreign-born and to smoke during the prenatal period, had more education, and had higher rates of overweight and obesity (S1 Table).

Approximately 12% of the subjects were SGA (118/1,003 = 11.8%). The average age at menarche was 147.5 months (12.3 years); 14% of participants had menarche before age 11 (Table 1). There was no difference in age at menarche between girls who were born SGA vs. those who were not born SGA. Half (50%) of the mothers were non-Hispanic Black, a quarter (27%) were Hispanic, and a third (33%) had less than a high school education. Almost two-thirds (64%) of the births were insured by Medicaid. A quarter (24%) of mothers were married, and 38% were cohabiting but not married at the time of the birth, reflecting the oversampling of nonmarital births in the FFCWB study. The sociodemographic characteristics of the sample reflect the strong associations between nonmarital childbearing, poverty, and racial-ethnic minority status in the United States [22, 23]. Further, 24% of the mothers were obese prior to pregnancy, 18% were overweight but not obese prior to pregnancy, and 23% smoked during pregnancy.

Compared to girls who were not SGA, those who were SGA were more likely to have mothers who were non-Hispanic Black (60% vs. 48%), had Medicaid-financed births (76% vs. 63%), and smoked during pregnancy (44% vs. 21%), and were less likely to have mothers who were Hispanic (19% vs. 28%) and married (14% vs. 26%).

### Age at menarche in months

In the unadjusted linear regression model, SGA was not significantly associated with earlier age at menarche (Table 2; OLS coef.: -1.27; CI: -4.54, 2.00). The estimate was similar in linear models that adjusted for maternal socioeconomic characteristics (OLS coef.: -1.34; CI: -4.64, 1.97) and that further adjusted for pre-pregnancy obesity, pre-pregnancy overweight, and prenatal smoking (OLS coef.: -1.18; CI: -4.55, 2.19).

**Table 2:** Ordinary least squares estimates of associations between SGA and age at menarche in months and between maternal characteristics and age at menarche.

	Unadjusted	Adjusted for maternal demographics	Adjusted for maternal demographics, pre-pregnancy obesity, pre-pregnancy overweight, and prenatal smoking
	OLS coefficient	OLS coefficient	OLS coefficient
	(95% CI)	(95% CI)	(95% CI)
SGA	-1.27	-1.34	-1.18
	(-4.54, 2.00)	(-4.64, 1.97)	(-4.55, 2.19)
Race-ethnicity			
Non-Hispanic White		ref	ref
Non-Hispanic Black		**-4.82**	**-4.50**
		**(-7.38, -2.26)**	**(-7.14, -1.86)**
Hispanic		**-6.70**	**-6.66**
		**(-9.79, -3.61)**	**(-9.82, -3.51)**
Other		-2.81	-2.84
		(-9.37, 3.74)	(-9.38, 3.70)
Education			
High school graduate		-1.37	-1.69
		(-3.73, 1.00)	(-4.07, 0.69)
Less than high school		ref	ref
Nativity			
US born		ref	
Foreign born		0.46	0.01
		(-2.82, 3.75)	(-3.29, 3.31)
Age			
< 20 years		0.27	-0.66
		(-3.80, 4.35)	(-4.75, 3.43)
20–34		0.72	0.32
		(-2.40, 3.85)	(-2.84, 3.49)
35+		ref	ref
Relationship with child’s father			
Married		1.70	1.49
		(-1.23, 4.64)	(-1.42, 4.40)
Cohabiting but not married		-0.47	-0.38
		(-2.78, 1.83)	(-2.69, 1.93)
Neither married nor cohabiting		ref	ref
Parity			
First birth		0.09	-0.25
		(-2.01, 2.19)	(-2.37, 1.87)
Second of higher order birth		ref	ref
Health insurance for birth			
Medicaid		1.49	1.45
		(-0.80, 3.79)	(-0.86, 3.75)
Other		ref	ref
Pre-pregnancy weight			
Obese			**-3.16**
			**(-5.78, -0.54)**
Overweight			**-2.90**
			**(-5.52, -0.28)**
Normal or underweight			ref
Prenatal smoking			
Any			-1.62
			(-4.15, 0.91)
None			ref

Notes: OLS = Ordinary Least Squares. SGA = small-for-gestational age = <10th percentile of sex-specific birth weight for gestational age. (N = 1,003). SGA was based on birth weight and last menstrual period (LMP)-based gestational age and compared to the national U.S. sex-specific distribution of LMP-based SGA from 1999–2000. All models included a binary indicator for missing data on age in menarche in months (i.e., the respondent reported her age at menarche but not in months in addition to her years of age) and the fully adjusted model included a binary indicator for missing pre-pregnancy weight. Statistically significant estimates at p < .05 are in bold.

In the fully adjusted model, having a non-Hispanic Black mother was associated with 4.5 months (.38 years) earlier menarche (OLS coef.: -4.50; CI: -7.14, -1.86), and having a Hispanic mother was associated with 6.7 months (.56 years) earlier menarche (OLS coef.: -6.66; CI: -9.82, -3.51). Maternal pre-pregnancy obesity (OLS coef.: -3.16; CI: -5.78, -0.54) and overweight (OLS coef.: -2.90; CI: -5.52, -0.28) were also independently associated with earlier menarche, by .26 and .24 years, respectively.

### Menarche before age 11

In the unadjusted logistic regression model, SGA was not significantly associated with the odds of menarche before age 11 (Table 3; OR 1.46; CI: 0.89, 2.42). The estimates were similar and never statistically significant when adjusting for maternal sociodemographic characteristics (AOR: 1.45; CI: 0.86, 2.46) or when further adjusting for pre-pregnancy obesity, pre-pregnancy overweight, and prenatal smoking (AOR: 1.47; CI: 0.85, 2.52). In the fully adjusted logistic regression model, maternal non-Hispanic Black race-ethnicity was associated with twice the odds (AOR: 2.06; CI: 1.12, 3.79), Hispanic ethnicity was associated with over two times the odds (AOR: 2.28; CI: 1.15, 4.49), and pre-pregnancy obesity was associated with 64% higher odds (AOR: 1.64; CI: 1.02, 2.62) of menarche before age 11.

**Table 3:** Logistic regression estimates of associations between SGA and menarche before age 11 and between maternal characteristics and menarche before age 11.

	Unadjusted	Adjusted for maternal demographics	Adjusted for maternal demographics, pre-pregnancy obesity, pre-pregnancy overweight, and prenatal smoking
	OR	AOR	AOR
	(95% CI)	(95% CI)	(95% CI)
SGA	1.46	1.45	1.47
	(0.89, 2.42)	(0.86, 2.46)	(0.85, 2.52)
Race-ethnicity			
Non-Hispanic White		ref	ref
Non-Hispanic Black		**2.21**	**2.06**
		**(1.22, 4.03)**	**(1.12, 3.79)**
Hispanic		**2.38**	**2.28**
		**(1.22, 4.66)**	**(1.15, 4.49)**
Other		2.46	2.42
		(0.82, 7.39)	(0.80, 7.32)
Education			
High school graduate		1.19	1.23
		(0.78, 1.82)	(0.80, 1.89)
Less than high school		ref	ref
Nativity			
US born		ref	ref
Foreign born		0.88	0.93
		(0.49, 1.59)	(0.52, 1.68)
Age			
< 20 years		1.70	1.90
		(0.69, 4.20)	(0.76, 4.72)
20–34		1.44	1.50
		(0.66, 3.15)	(0.68, 3.27)
35+		ref	ref
Relationship with child’s father			
Married		0.77	0.77
		(0.43, 1.38)	(0.43, 1.38)
Cohabiting but not married		1.10	1.10
		(0.75, 1.63)	(0.74, 1.63)
Neither married not cohabiting		ref	ref
Parity			
First birth		0.97	1.02
		(0.66, 1.43)	(0.69, 1.52)
Second or higher order birth		ref	ref
Health insurance for birth			
Medicaid		0.87	0.88
		(0.57, 1.34)	(0.57, 1.36)
Other		ref	ref
Pre-pregnancy weight			
Obese			**1.64**
			**(1.02, 2.62)**
Overweight			1.55
			(0.93, 2.57)
Normal or underweight			ref
Prenatal smoking			
Any			1.09
			(0.70, 1.69)
None			ref

Notes: AOR = adjusted odds ratio. OR = odds ratio. SGA = small-for-gestational age = <10th percentile of sex-specific birth weight for gestational age. (N = 1,027). SGA was based on birth weight and LMP-based gestational age and compared to the national U.S. sex-specific distribution of LMP-based SGA from 1999–2000. Fully adjusted models included a binary indicator for missing data on pre-pregnancy weight. Statistically significant estimates at p < .05 are in bold.

### Supplemental analyses

In models that used a 25^th^ percentile cutoff to define SGA, SGA was not significantly associated with age at menarche in months or menarche before age 11 (not shown). Models that alternatively included a control for gestational age in weeks, restricted the sample to full-term births (37–41 weeks), restricted the sample to children who weighed > 2500 grams at birth, restricted the sample for both outcomes to girls who reported month of menarche as well as the year, used the smaller sample of 1,003 in the logistic regression models, restricted the sample to cases that had non-missing data on pre-pregnancy weight, or used inverse probability weights to account for missing observations all produced estimates that were very similar to those in Tables 2 and 3 (not shown). In supplementary models that interacted weight gain between birth and age 1 with SGA, we found no significant interactions. However, weight gain was associated with earlier menarche in those models (e.g., OLS coeff: -.75; CI: -1.36, -0.15) in the fully adjusted model for age at menarche in months and AOR: 1.13; CI: 1.02, 1.26 in the fully adjusted model for menarche before age 11) (not shown in tables).

## Discussion

In this prospective study of a population-based U.S. birth cohort that included large proportions of subjects with racial-ethnic minority status and low socioeconomic status, SGA was not significantly associated with earlier age at menarche. As such, this manuscript contributes valuable data points to the literature that has found mixed results for associations between SGA and pubertal development. However, maternal Black race, Hispanic ethnicity, and pre-pregnancy obesity had strong independent associations with daughters’ earlier age at menarche and with menarche before age 11 in this population. For example, girls with non-Hispanic Black mothers had menarche about a third of a year earlier than those with non-Hispanic White mothers, girls with Hispanic mothers had menarche over half a year earlier than those with non-Hispanic White mothers, and girls whose mothers were obese or overweight had menarche about a quarter of a year earlier than those whose mothers were not obese or overweight. These findings corroborate evidence from more racially-ethnically homogeneous populations of associations between both minority race-ethnicity and maternal obesity and earlier menarche in offspring [32–34].

It should be noted that the contributions of race-ethnicity and BMI are challenging to disentangle as those factors are integrated in a complex manner; for example, racial-ethnic compositions of communities have been associated with individual-level BMI independent of individual-level race-ethnicity [35]. A multi-center U.S. study found that child BMI, which is associated with maternal BMI [36], had a stronger association with age at menarche than race-ethnicity did [37], and another recent study in California found that childhood obesity was associated with earlier menarche, but the magnitude of the association varied by race-ethnicity [38]. In moving towards race-conscious medicine, the relationship between race-ethnicity and obesity is understood to be a consequence of environmental racism rather than race pathologization [39]. For example, youth obesity is known to be associated with lower socioeconomic status and food insecurity [40], and early puberty or menarche is known to be associated with early life stress and potential environmental chemical exposures from marketed products [41–43]. All of these adverse exposures are disproportionately experienced by members of racial-ethnic minority groups [39]. Although we studied a population that over-sampled non-marital births and were able to control for many socioeconomic factors, we could not account for all potentially relevant environmental, social, and perhaps genetic or epigenetic factors in our study.

As discussed, SGA or low birth weight models propose that “catch-up growth” leads to increased adiposity and subsequent earlier puberty [44]. Adiposity may be defined in terms of body composition, biochemical changes, abdominal obesity, or weight and weight velocity. In the Danish cohort noted earlier, the absence or presence of catch-up growth in SGA children significantly contributed to age at menarche, such that those without catch-up growth had later menarche [19]. Further, a multi-center U.S. cohort study showed that independent of AGA or SGA status, more significant weight gain in early childhood was associated with earlier menarche and pubertal development [37]. Interestingly, one study found that increased total body fat led to slower progression of breast development but faster achievement of menarche [45]. Unfortunately, we were unable to fully incorporate the potential role of the child’s weight in this study because the children’s weights and heights were not consistently measured in the FFCWB study. However, our supplementary analyses that interacted SGA with first-year weight gain suggested no interactive associations, although weight gain was independently associated with earlier menarche. Additionally, we could not account for maternal age at menarche, which has been associated with changes in offspring BMI [46]. Our study instead focused on maternal factors known to be associated with elevated child BMI, such as race-ethnicity [47] and maternal pre-pregnancy obesity [36].

Moreover, increased adiposity is a complex phenomenon studied in SGA models, and ideally, it would not be evaluated solely using indirect measures such as BMI. A longitudinal cohort study using multiple adiposity indicators including BMI, waist circumference, and body composition found that increased prepubertal adiposity was associated with an increased risk of earlier puberty onset [48]. Also, adiposity measured directly as body composition had a stronger relationship to earlier puberty onset than indirect measures (BMI or waist circumference) [48]. Biochemical characteristics of increased adiposity were studied in SGA girls, showing they were more likely to develop hyperinsulinism, hypoadiponectinemia, hyperleptinemia, dyslipidemia, lower sex hormone binding globulin, higher dehydroepiandrosterone sulfate, and advanced bone maturation [13]. We could not distinguish between adiposity measured from body composition versus BMI using our data.

Strengths of our study include the population-based U.S. cohort and inclusion of rich control variables, including maternal race-ethnicity, other sociodemographic characteristics, and maternal pre-pregnancy body weight. Because the FFCWB study oversampled non-marital births by design, it is not representative of the U.S. population. Indeed, 11.7% of the subjects in our sample were SGA, which is expectedly higher than the national rate of 10%. However, children of unmarried parents represent a policy-relevant population in the U.S. because of their disproportionate risks of developmental problems as a result of socioeconomic risk factors, including racial-ethnic minority status and poverty.

Although we found no significant associations between SGA and menarche before age 11, the magnitudes of the ORs were not trivial, and it is possible that with a larger sample the associations would have been statistically significant. Other limitations include loss to follow-up; the mothers of the children in our analysis sample were less likely to be foreign-born and to smoke during the prenatal period and had higher education and rates of overweight and obesity compared to mothers of children not in our analysis sample, which could bias the estimates, although the expected direction of bias is not clear. Also, our study data uses self-reported age at menarche, and many subjects only knew their approximate age in years instead of a specific date. Although menarche before age 11 is a frequently used cutoff to define early menarche, we could not use stricter cutoffs, such as before age 10, since too few girls in our sample experienced menarche within that age range for meaningful analysis. There is evidence that the age of puberty is decreasing for males as it has been for females [49], but we were not able to investigate associations between SGA and puberty in males with our data since there was no survey marker obtained for male puberty. In addition, causality cannot be firmly established in any observational study.

In a national U.S. birth cohort that included large proportions of subjects with racial-ethnic minority status, SGA status was not associated with girls’ age at menarche, but maternal race-ethnicity and pre-pregnancy obesity were strongly and independently associated with daughters’ earlier age at menarche. Additional insight can be gained from future prospective studies using body composition analysis to measure adiposity and explore the relationship between SGA, race-ethnicity, and age at menarche.

## Supporting information

S1 Table Comparison of analysis sample to singleton female births not in the sample. Notes: Calculations are based on sample used for analyses of age at menarche in months. P-values are for differences between those in the sample for age at menarche in months and singleton females not in that sample at the 5% level using a two-tailed t-test (statistically significant p-values in bold).
(DOCX)
