# Supplementary material for: Small for gestational age and age at menarche in a contemporary population-based U.S. sample
Source: PLoS One. 2024 Sep 6;19(9):e0309363. doi: 10.1371/journal.pone.0309363 (PMC11379201; doi:10.1371/journal.pone.0309363)
Supplement: S1 Table — Notes: Calculations are based on sample used for analyses of age at menarche in months. P-values are for differences between those in the sample for age at menarche in months and singleton females not in that sample at the 5% level using a two-tailed t-test (statistically significant p-values in bold). (DOCX) [file pone.0309363.s001.docx]

**S1 Table. Comparison of analysis sample to singleton female births not in the sample.**

|  | | **In sample** | **Not in sample** | **p-value** |
| --- | --- | --- | --- | --- |
| **Maternal sociodemographic characteristics** | |  |  |  |
| Race-ethnicity | |  |  |  |
|  | Non-Hispanic White | 0.21 | 0.21 | 0.78 |
|  | Non-Hispanic Black | 0.50 | 0.46 | 0.06 |
|  | Hispanic | 0.27 | 0.29 | 0.27 |
|  | Other race | 0.03 | 0.05 | 0.08 |
| Education | |  |  |  |
|  | High school graduate | 0.67 | 0.62 | **0.01** |
|  | Less than high school | 0.33 | 0.38 | **0.01** |
| Nativity | |  |  |  |
|  | US born | 0.86 | 0.81 | **<0.01** |
|  | Foreign born | 0.14 | 0.19 | **<0.01** |
| Age, mean years | |  |  |  |
|  | < 20 | 0.17 | 0.16 | 0.33 |
|  | 20–34 | 0.74 | 0.73 | 0.71 |
|  | 35+ | 0.09 | 0.11 | 0.08 |
| Relationship with child’s father | |  |  |  |
|  | Married | 0.24 | 0.24 | 0.87 |
|  | Cohabiting but not married | 0.38 | 0.37 | 0.87 |
|  | Neither married nor cohabiting | 0.38 | 0.39 | 0.77 |
| Parity | |  |  |  |
|  | First birth | 0.39 | 0.36 | 0.19 |
|  | Higher-order birth | 0.61 | 0.64 | 0.19 |
| Health insurance for birth | |  |  |  |
|  | Medicaid | 0.64 | 0.67 | 0.13 |
|  | Other | 0.36 | 0.33 | 0.13 |
| **Other maternal variables** | |  |  |  |
| Pre-pregnancy weight | |  |  |  |
|  | Obese | 0.24 | 0.11 | **<0.01** |
|  | Overweight (but not obese) | 0.18 | 0.10 | **<0.01** |
|  | Normal or underweight | 0.58 | 0.79 | **<0.01** |
| Prenatal smoking | |  |  |  |
|  | Any | 0.23 | 0.38 | **<0.01** |
|  | None | 0.77 | 0.62 | **<0.01** |
| N | | 1,003 | 1,292 |  |

Notes: Calculations are based on sample used for analyses of age at menarche in months. P-values are for differences between those in the sample for age at menarche in months and singleton females not in that sample at the 5% level using a two-tailed t-test (statistically significant p-values in bold).
