# Supplementary material for: Small for gestational age and age at menarche in a contemporary population-based U.S. sample
Source: PLoS One. 2024 Sep 6;19(9):e0309363. doi: 10.1371/journal.pone.0309363 (PMC11379201; doi:10.1371/journal.pone.0309363)
Supplement: Attachment — Submitted filename: Response to Reviewers.docx [file pone.0309363.s002.docx]

**Responses regarding journal requirements:** (our responses are indicated by the bulleted arrow)

When submitting your revision, we need you to address these additional requirements.

1. Please ensure that your manuscript meets PLOS ONE's style requirements, including those for file naming. The PLOS ONE style templates can be found at

https://journals.plos.org/plosone/s/file?id=wjVg/PLOSOne_formatting_sample_main_body.pdf

and

https://journals.plos.org/plosone/s/file?id=ba62/PLOSOne_formatting_sample_title_authors_affiliations.pdf

- We ensured that our manuscript meets PLOS ONE’s style requirements.

2. Thank you for stating the following financial disclosure:

“Support for this research was provided by award UL1TR003017 from the National Center for Advancing Translational Sciences, a component of the National Institutes of Health and grant 74260 from the Robert Wood Johnson Foundation through its support of the Child Health Institute of New Jersey.”

Please state what role the funders took in the study. If the funders had no role, please state: "The funders had no role in study design, data collection and analysis, decision to publish, or preparation of the manuscript."

If this statement is not correct you must amend it as needed. Please include this amended Role of Funder statement in your cover letter; we will change the online submission form on your behalf.

- We added the suggested statement to the financial disclosure and included the revised financial disclosure in the cover letter, as directed.

3. We note that you have indicated that there are restrictions to data sharing for this study. PLOS only allows data to be available upon request if there are legal or ethical restrictions on sharing data publicly. For more information on unacceptable data access restrictions, please see http://journals.plos.org/plosone/s/data-availability#loc-unacceptable-data-access-restrictions.

Before we proceed with your manuscript, please address the following prompts:

a) If there are ethical or legal restrictions on sharing a de-identified data set, please explain them in detail (e.g., data contain potentially identifying or sensitive patient information, data are owned by a third-party organization, etc.) and who has imposed them (e.g., a Research Ethics Committee or Institutional Review Boar d, etc.). Please also provide contact information for a data access committee, ethics committee, or other institutional body to which data requests may be sent.

b) If there are no restrictions, please upload the minimal anonymized data set necessary to replicate your study findings to a stable, public repository and provide us with the relevant URLs, DOIs, or accession numbers. For a list of recommended repositories, please see

https://journals.plos.org/plosone/s/recommended-repositories. You also have the option of uploading the data as Supporting Information files, but we would recommend depositing data directly to a data repository if possible.

We will update your Data Availability statement on your behalf to reflect the information you provide.

[Note: HTML markup is below. Please do not edit.]

- We included the following data availability statement in our cover letter:

*We used third-party data that are available to the public through a restricted data use contact from the Future of Families & Child Wellbeing Study at Princeton University. Details about the application process can be found at:* [*https://ffcws.princeton.edu/restricted*](https://ffcws.princeton.edu/restricted)*.*

**Responses to Reviewers**

- We thank the reviewers for the very helpful comments.
- Please note that in reanalyzing the data, we found that we were able to include a slightly larger number of cases in our analyses. This did not change any findings or interpretations. We also now focus exclusively on the measure of SGA that was based on LMP-based gestational age.

**Reviewer #1:** The article entitled “Small for gestational age and age at menarche in a contemporary population-based US sample” tested the hypothesis that SGA girls experience earlier menarche. Prior reports of racially homogenous populations have yielded mixed results. This analysis of a largely minority racial/ethnic population showed that maternal factors such as race/ethnicity and obesity influenced age of menarche, rather than SGA status. The data provides valuable information to add to our understanding of factors that influence pubertal onset.

Specific comments:

1. Please provide more context to the FFCWB study, that may explain the demographic of the study population.

- We now include the following paragraph on page 5, line 100:

  *The FFCWB cohort was population-based because it used a probability sample of cities, hospitals, and births within hospitals. Non-marital births were oversampled by design; that is, marital births were no longer included when hospital-specific quotas were reached. The reason for the oversampling was that unmarried parents and their children had been underrepresented in national U.S. datasets. About three-fourths of the mothers in the study were unmarried when they gave birth [25,26]. Because non-marital childbearing is strongly associated with poverty in the U.S., as is racial/ethnic minority status, the FFCWB sample had high fractions of poor, Black, and Hispanic parents.*

2. The paragraph describing SGA (lines 113 to 125) was confusing. Line 114 – please clarify. Are the authors referring to determination of the gestational age? It’s also unclear why two different methods were needed to define SGA. subjects used the LMP vs the records-based measure.

- We edited and clarified the paragraph describing SGA (page 6, line 116). We had been referring to the determination of gestational age, but that should be clear in the revised text, which now reads as follows:

*SGA was defined as <10^th^ percentile of sex-specific birth weight for gestational age. Gestational age was calculated as the number of completed weeks between the date of the first day of the mother’s last menstrual period (LMP) and the child’s birthday. The respondent’s birth weight for gestational age was compared to the national U.S. sex-specific reference distribution of LMP-based SGA from 1999–2000[28] when most of the FFCWB sample members were born.*

- Please note that we now focus exclusively on the measure of SGA that used LMP-based gestational age.

3. Lines 177/178: Is it the controls that were limited to births that were NOT low birthweight?

- We clarified the text to indicate that we restricted the sample to children that weighed more than 2500 grams at birth in those analyses.

4.	In Results, I recommend describing the difference in race/ethnicity between SGA vs not SGA.

- Please see the following sentence on line 212 of the revised paper (bolding is for emphasis here):

***Compared to girls who were not SGA, those who were SGA were more likely to have mothers who were non-Hispanic Black (60% vs. 48%),*** *had Medicaid-financed births (76% vs. 63%), and smoked during pregnancy (44% vs. 21%),* ***and were less likely to have mothers who were Hispanic (19% vs. 28%) and married (14% vs. 26%).***

4. Substantial discussion was given to the effect of obesity on early menarche but there was almost no discussion given for the effect of race/ethnicity on the same. Please elaborate on the current understanding of the relationship between race/ethnicity and pubertal timing (including if relevant, early childhood stress, environmental racism, Pediatrics (2022) 150 (2): e2021055595. https://doi.org/10.1542/peds.2021-055595).

- We elaborated on the current understanding of the relationship between race/ethnicity and pubertal timing, bringing in the suggested article (line 288). We agree that this relationship was underdiscussed in the previous version of the paper.

**Reviewer #2:** The Authors hereby report the menarche onset in a pediatric cohort of small for gestational age females. The data are of interest and the cohort has a quite adequate dimension. There are two main concerns about this paper:

1. Table 1 should be moved into the Results sections, p column should be added in the right and not reported in legend

- Done.

2. Why Authors chose 11 years old as a cut off for menarche? Precious menarche is considered as before 10 years-old, thus all considerations about whether SGA newborn females have precocious menarche more frequently rather than non-SGA should be done considering the 10 years-old cut-off.

- As we now indicate on line 51, no standard cut-off has been used in the literature; cut-offs have ranged from 10.5 to 12 and age 11 has been used in other studies (line 132). That said, we estimated supplementary models with age 10 as the cutoff (menarche before age 10), but with only 38 girls in our sample with early menarche defined this way, we were not able to estimate adjusted models. In the unadjusted models, SGA was not significantly associated with age in months at menarche or early menarche. We now point to not being able to use a more stringent cut-off as a limitation of our study (line 342).
- Also, please note that we now focus exclusively on the measure of SGA that uses LMP-based gestational age.

**Reviewer #3**: Well written with statistics supporting the results. Also the arguments and rationale is well supported with references. Population sample is also presentation of contemporary US population. Overall good addition to the current literature.

- Thank you.

**Reviewer #4:** General comments: Sources of variation in age at menarche are a useful focus of investigation. The authors did not find associations between SGA and age at menarche. They did identify a number of other maternal factors related to age at menarche. The data are overall presented clearly, though there are some opportunities to improve formatting and readability suggested below, as well as questions regarding some of the statements (e.g., when the authors compare the value of two coefficients).

1. Did the authors have any other health information about the girls at follow up (e.g., BMI) that also could yield more information, even if they did not have information about catch-up growth patterns?

- We now report on supplemental results from models that interacted SGA and weight gain between birth and one year (line 262):

*In supplementary models that interacted weight gain between birth and age 1 with SGA, we found no significant interactions. However, weight gain was associated with earlier menarche in those models (e.g., OLS coeff: -.75; CI: -1.36, -0.15) in the fully adjusted model for age at menarche in months and AOR: 1.13; CI: 1.02, 1.26 in the fully adjusted model for menarche before age 11) (not shown in tables).*

- Unfortunately, we do not have better health data to use for this supplementary analysis.

2. Sex differences are also interesting – can the authors also speculate as to the differences, if any, between maternal factors and pubertal outcomes in female infants as compared to male infants?

- We now point to not being able to study linkages between SGA and puberty for males as a limitation of our study (line 344).

3. Abstract: The database consisting of mostly of non-marital births may not be representative of the U.S. population; the authors should comment in the Discussion on how their results may differ. That said, with this current focus, the database may teach us more about children deserving of additional study.

- We now indicate in the abstract that the sample consisted of mostly non-marital births and highlight that it represents a policy-relevant population in the U.S. We elaborate on this point when commenting on the strengths and limitations of the study in the Discussion (line 326).

4. The authors describe “early menarche” as before age 11 years. Care should be taken to distinguish between “early menarche” (variation of typical) and “premature menarche” (implying the possibility of pathology).

- We now make this distinction (line 53).

5. If the difference was not statistically significant, then the authors should not write “SGA was associated with slightly earlier menarche” which implies some meaningful difference. Instead, they can write, e.g., “We did not detect a difference.”

- We changed the wording throughout to avoid such language.

6. The authors conclude that maternal non-Hispanic black race-ethnicity, Hispanic ethnicity, and pre-pregnancy obesity were associated with earlier age at menarche. If these factors related to SGA itself, could there be evidence of mediation?

- This was an interesting comment. We ran supplementary models that did not include SGA to assess how much of a difference including SGA in the models affected the estimates for maternal non-Hispanic black race-ethnicity, Hispanic ethnicity, and pre-pregnancy obesity. We found that the estimates for all of these factors were very similar to those shown in the main models, suggesting no mediation. We could not find a straightforward way to motivate these supplementary analyses in the “supplementary analyses” section, so we ended up not reporting on these interesting supplementary results.

7. Early menarche is defined as before age 10.5y (line 47) but before age 11y in this manuscript; it is worth distinguishing the reasons.

- As we now indicate on line 51, no standard cut-off has been used in the literature; cut-offs have ranged from 10.5 to 12 and age 11 has been used in other studies (line 132). That said, we estimated supplementary models with age 10 as the cutoff (menarche before age 10), but with only 38 girls in our sample with early menarche defined this way, we were not able to estimate adjusted models. In the unadjusted models, SGA was not significantly associated with age in months at menarche or early menarche. We now point to not being able to use a more stringent cut-off as a limitation of our study (line 342).

8. Line 52 – The implied causal association between early menarche and mental health problems and antisocial behaviors is potentially fraught – adverse socioeconomic circumstances might explain both. The authors should either outline more about what is known about causality and/or make sure to differentiate.

- We now make this important point as follows on line 56:

  *Further, girls with early menarche are more likely to experience depressive symptoms and engage in health risk behaviors that adversely impact health in adulthood[7], although it has not been established whether those associations are confounded by socioeconomic factors.*

9. There is a fair number of missing measurements (859 out of a starting 2,295) – can the authors comment on any differences between those newborns with versus without available data (presumably maternal information at enrollment may be available)?

- *Done. This information is now presented in S1 Table.*

10. What do the authors make of the finding that 10-11.6% of their sample was SGA (depending on measurement technique used)? This proportion is aligned with the expected proportion of <10% in the general population, even though their sample was designed to be enriched in individuals who might be expected to be at higher risk to have infants who are SGA?

- We now focus exclusively on the analyses based on LMP-based gestational age. Using that measure, 11.7% of our sample was SGA and we highlight that this figure is expectedly higher than in the general population because of the largely disadvantaged population we studied (line 329).

11. Line 152: BMI is weight in kg divided by height in meters (not centimeters) squared

- We corrected this typographical error.

12. Present the differences between those included versus excluded from the sample in table form, i.e., include these details, since there were age and income differences.

- Done. We now present this information in S1 Table for all of the variables that are in our analyses.

13. Table 1 – should be presented in Results, and since this Table refers to the analysis cohort only, should be discussed after the in versus out of cohort.

- Done.

14. Age is reported in months to 2 significant digits. Suggest to 1 or no decimal points, and reporting 95% confidence intervals. See PMID 25877157. Suggest statistically significant differences between SGA and non-SGA be indicated in bold text.-Formatting could be improved, e.g., to indicate explicitly categories for age.

- We made these changes.

15. Again, the nominal difference of 1 month in Line 96 should not be presented as a “difference” if not significant; if the authors choose to show this nominal difference, they should also show the 95% CI for the difference in the text (not just the table). Suggest revising number of decimal points.

- We made wording changes consistent with this suggestion throughout and also changed the number of decimal points.

16. Line 214, same comment. Instead, they did not detect a difference.

- We made wording changes consistent with this suggestion throughout.

17. Line 219 – “the association was even larger” – there are post hoc statistical tests that can discern if the coefficients are truly statistically different from each other, which given substantial overlap in confidence intervals they may not be. Suggest revising.

- We no longer refer to relative strength of associations between different factors and the outcomes.

18. Table 2 could also be formatted such that categories (e.g., age, race, are more explicitly grouped would improve readability). Bold text for statistically significant associations would be helpful. Similar feedback for Table 3.

- We made these changes.

19. Discussion: Line 264 – although the coefficient is nominally larger, there are specific statistical tests that are used to determine whether the coefficients are statistically different –were these done?

- We no longer refer to relative strength of associations between different factors and the outcomes.

20. Overall, the results are situated in the existing literature. The Discussion would be enhanced by some more mechanistic insights from the literature underlying the epidemiologic associations observed.

- We now provide insights from the literature regarding potential mechanisms underlying the observed associations between both race-ethnicity and maternal obesity and the timing of menarche (line 281).
