# Supplementary material for: Small for gestational age and age at menarche in a contemporary population-based U.S. sample
Source: PLoS One. 2024 Sep 6;19(9):e0309363. doi: 10.1371/journal.pone.0309363 (PMC11379201; doi:10.1371/journal.pone.0309363)
Supplement: Attachment — Submitted filename: Response to Reviewers.docx [file pone.0309363.s003.docx]

1. Line 248/249: As there was no statistically significant difference in menarche age between SGA and non-SGA, the statement should be modified to state “There was no difference in early menarche between girls who were SGA vs. non-SGA.”

- The line number listed by the reviewer did not correlate to a relevant sentence. But we noted a similar statement earlier in our paper. We made this change (line 197-198) that now reads:

*There was no difference in age at menarche between girls who were born SGA vs. those who were not born SGA.*

2. Please confirm in Table 2, for Adjusted maternal demographics, for Hispanic race ethnicity, if the upper CI is 3.61 or -3.61.

- Thank you for catching this typo. The upper CI should be -3.61. We made this change.
