# Supplementary material for: Small for gestational age and age at menarche in a contemporary population-based U.S. sample
Source: PLoS One. 2024 Sep 6;19(9):e0309363. doi: 10.1371/journal.pone.0309363 (PMC11379201; doi:10.1371/journal.pone.0309363)
Supplement: Peer review history

# Peer review: Decision Letter 0

**DOI:** 10.1371/journal.pone.0309363.r001

**Authors:** Inge Roggen

17 May 2024

PONE-D-24-13471Small for gestational age and age at menarche in a contemporary population-based U.S. samplePLOS ONE

Dear Dr. Sabu,

Thank you for submitting your manuscript to PLOS ONE. After careful consideration, we feel that it has merit but does not fully meet PLOS ONE’s publication criteria as it currently stands. Therefore, we invite you to submit a revised version of the manuscript that addresses the points raised during the review process.

Please find the reviewer's remarques below.

Please submit your revised manuscript by Jul 01 2024 11:59PM. If you will need more time than this to complete your revisions, please reply to this message or contact the journal office at plosone@plos.org. When you're ready to submit your revision, log on to https://www.editorialmanager.com/pone/ and select the 'Submissions Needing Revision' folder to locate your manuscript file.

Please include the following items when submitting your revised manuscript:

- A rebuttal letter that responds to each point raised by the academic editor and reviewer(s). You should upload this letter as a separate file labeled 'Response to Reviewers'.
- A marked-up copy of your manuscript that highlights changes made to the original version. You should upload this as a separate file labeled 'Revised Manuscript with Track Changes'.
- An unmarked version of your revised paper without tracked changes. You should upload this as a separate file labeled 'Manuscript'.

If you would like to make changes to your financial disclosure, please include your updated statement in your cover letter. Guidelines for resubmitting your figure files are available below the reviewer comments at the end of this letter.

If applicable, we recommend that you deposit your laboratory protocols in protocols.io to enhance the reproducibility of your results. Protocols.io assigns your protocol its own identifier (DOI) so that it can be cited independently in the future. For instructions see: https://journals.plos.org/plosone/s/submission-guidelines#loc-laboratory-protocols. Additionally, PLOS ONE offers an option for publishing peer-reviewed Lab Protocol articles, which describe protocols hosted on protocols.io. Read more information on sharing protocols at https://plos.org/protocols?utm_medium=editorial-email&utm_source=authorletters&utm_campaign=protocols.

We look forward to receiving your revised manuscript.

Kind regards,

Inge Roggen, M.D., Ph.D.

Academic Editor

PLOS ONE

Journal requirements:

When submitting your revision, we need you to address these additional requirements.

1. Please ensure that your manuscript meets PLOS ONE's style requirements, including those for file naming. The PLOS ONE style templates can be found at

https://journals.plos.org/plosone/s/file?id=wjVg/PLOSOne_formatting_sample_main_body.pdf and

https://journals.plos.org/plosone/s/file?id=ba62/PLOSOne_formatting_sample_title_authors_affiliations.pdf

2. Thank you for stating the following financial disclosure:

“Support for this research was provided by award UL1TR003017 from the National Center for Advancing Translational Sciences, a component of the National Institutes of Health and grant 74260 from the Robert Wood Johnson Foundation through its support of the Child Health Institute of New Jersey.”

Please state what role the funders took in the study.  If the funders had no role, please state: "The funders had no role in study design, data collection and analysis, decision to publish, or preparation of the manuscript."

If this statement is not correct you must amend it as needed.

Please include this amended Role of Funder statement in your cover letter; we will change the online submission form on your behalf.

3. We note that you have indicated that there are restrictions to data sharing for this study. PLOS only allows data to be available upon request if there are legal or ethical restrictions on sharing data publicly. For more information on unacceptable data access restrictions, please see http://journals.plos.org/plosone/s/data-availability#loc-unacceptable-data-access-restrictions.

Before we proceed with your manuscript, please address the following prompts:

a) If there are ethical or legal restrictions on sharing a de-identified data set, please explain them in detail (e.g., data contain potentially identifying or sensitive patient information, data are owned by a third-party organization, etc.) and who has imposed them (e.g., a Research Ethics Committee or Institutional Review Board, etc.). Please also provide contact information for a data access committee, ethics committee, or other institutional body to which data requests may be sent.

b) If there are no restrictions, please upload the minimal anonymized data set necessary to replicate your study findings to a stable, public repository and provide us with the relevant URLs, DOIs, or accession numbers. For a list of recommended repositories, please see

https://journals.plos.org/plosone/s/recommended-repositories. You also have the option of uploading the data as Supporting Information files, but we would recommend depositing data directly to a data repository if possible.

We will update your Data Availability statement on your behalf to reflect the information you provide.

[Note: HTML markup is below. Please do not edit.]

Reviewers' comments:

Reviewer's Responses to Questions

**Comments to the Author**

1. Is the manuscript technically sound, and do the data support the conclusions?

The manuscript must describe a technically sound piece of scientific research with data that supports the conclusions. Experiments must have been conducted rigorously, with appropriate controls, replication, and sample sizes. The conclusions must be drawn appropriately based on the data presented. 

Reviewer #1: Yes

Reviewer #2: Yes

Reviewer #3: Yes

Reviewer #4: Partly

**********

2. Has the statistical analysis been performed appropriately and rigorously? 

Reviewer #1: Yes

Reviewer #2: Yes

Reviewer #3: Yes

Reviewer #4: Yes

**********

3. Have the authors made all data underlying the findings in their manuscript fully available?

The PLOS Data policy requires authors to make all data underlying the findings described in their manuscript fully available without restriction, with rare exception (please refer to the Data Availability Statement in the manuscript PDF file). The data should be provided as part of the manuscript or its supporting information, or deposited to a public repository. For example, in addition to summary statistics, the data points behind means, medians and variance measures should be available. If there are restrictions on publicly sharing data—e.g. participant privacy or use of data from a third party—those must be specified.

Reviewer #1: Yes

Reviewer #2: Yes

Reviewer #3: No

Reviewer #4: Yes

**********

4. Is the manuscript presented in an intelligible fashion and written in standard English?

PLOS ONE does not copyedit accepted manuscripts, so the language in submitted articles must be clear, correct, and unambiguous. Any typographical or grammatical errors should be corrected at revision, so please note any specific errors here.

Reviewer #1: Yes

Reviewer #2: Yes

Reviewer #3: Yes

Reviewer #4: Yes

**********

5. Review Comments to the Author

Please use the space provided to explain your answers to the questions above. You may also include additional comments for the author, including concerns about dual publication, research ethics, or publication ethics. (Please upload your review as an attachment if it exceeds 20,000 characters)

Reviewer #1: The article entitled “Small for gestational age and age at menarche in a contemporary population-based US sample” tested the hypothesis that SGA girls experience earlier menarche. Prior reports of racially homogenous populations have yielded mixed results. This analysis of a largely minority racial/ethnic population showed that maternal factors such as race/ethnicity and obesity influenced age of menarche, rather than SGA status. The data provides valuable information to add to our understanding of factors that influence pubertal onset.

Specific comments:

1. Please provide more context to the FFCWB study, that may explain the demographic of the study population.

2. The paragraph describing SGA (lines 113 to 125) was confusing. Line 114 – please clarify. Are the authors referring to determination of the gestational age? It’s also unclear why two different methods were needed to define SGA. subjects used the LMP vs the records-based measure.

3. Lines 177/178: Is it the controls that were limited to births that were NOT low birth weight?

4. In Results, I recommend describing the difference in race/ethnicity between SGA vs not SGA.

5. Substantial discussion was given to the effect of obesity on early menarche but there was almost no discussion given for the effect of race/ethnicity on the same. Please elaborate on the current understanding of the relationship between race/ethnicity and pubertal timing (including if relevant, early childhood stress, environmental racism, Pediatrics (2022) 150 (2): e2021055595. https://doi.org/10.1542/peds.2021-055595).

Reviewer #2: The Authors hereby report the menarche onset in a pediatric cohort of small for gestational age females. The data are of interest and the cohort has a quite adequate dimension.

There are two main concerns about this paper:

- Table 1 should be moved into the Results sections, p column should be added in the right and not reported in legend

- Why Authors chose 11 years old as a cut off for menarche? Precious menarche is considered as before 10 years-old, thus all considerations about whether SGA newborn females have precocious menarche more frequently rather than non-SGA should be done considering the 10 years-old cut-off.

Reviewer #3: Well written with statistics supporting the results. Also the arguments and rationale is well supported with references . Population sample is also presentation of contemporary US population. Overall good addition to the current literature.

Reviewer #4: General comments:

Sources of variation in age at menarche are a useful focus of investigation. The authors did not find associations between SGA and age at menarche. They did identify a number of other maternal factors related to age at menarche. The data are overall presented clearly, though there are some opportunities to improve formatting and readability suggested below, as well as questions regarding some of the statements (e.g., when the authors compare the value of two coefficients).

Did the authors have any other health information about the girls at follow up (e.g., BMI) that also could yield more information, even if they did not have information about catch-up growth patterns?

Sex differences are also interesting – can the authors also speculate as to the differences, if any, between maternal factors and pubertal outcomes in female infants as compared to male infants?

Section-specific comments:

Abstract:

The database consisting of mostly of non-marital births may not be representative of the U.S. population; the authors should comment in the Discussion on how their results may differ. That said, with this current focus, the database may teach us more about children deserving of additional study.

The authors describe “early menarche” as before age 11 years. Care should be taken to distinguish between “early menarche” (variation of typical) and “premature menarche” (implying the possibility of pathology).

If the difference was not statistically significant, then the authors should not write “SGA was associated with slightly earlier menarche” which implies some meaningful difference. Instead, they can write, e.g., “We did not detect a difference.”

The authors conclude that maternal non-Hispanic black race-ethnicity, Hispanic ethnicity, and pre-pregnancy obesity were associated with earlier age at menarche. If these factors related to SGA itself, could there be evidence of mediation?

Introduction:

Early menarche is defined as before age 10.5y (line 47) but before age 11y in this manuscript; it is worth distinguishing the reasons.

Line 52 – The implied causal association between early menarche and mental health problems and antisocial behaviors is potentially fraught – adverse socioeconomic circumstances might explain both. The authors should either outline more about what is known about causality and/or make sure to differentiate.

Methods:

There is a fair number of missing measurements (859 out of a starting 2,295) – can the authors comment on any differences between those newborns with versus without available data (presumably maternal information at enrollment may be available)?

What do the authors make of the finding that 10-11.6% of their sample was SGA (depending on measurement technique used)? This proportion is aligned with the expected proportion of <10% in the general population, even though their sample was designed to be enriched in individuals who might be expected to be at higher risk to have infants who are SGA?

Line 152: BMI is weight in kg divided by height in meters (not centimeters) squared

Results:

Present the differences between those included versus excluded from the sample in table form, i.e., include these details, since there were age and income differences.

Table 1 – should be presented in Results, and since this Table refers to the analysis cohort only, should be discussed after the in versus out of cohort.

-Age is reported in months to 2 significant digits. Suggest to 1 or no decimal points, and reporting 95% confidence intervals. See PMID 25877157. Suggest statistically significant differences between SGA and non-SGA be indicated in bold text.

-Formatting could be improved, e.g., to indicate explicitly categories for age.

Again, the nominal difference of 1 month in Line 96 should not be presented as a “difference” if not significant; if the authors choose to show this nominal difference, they should also show the 95% CI for the difference in the text (not just the table). Suggest revising number of decimal points. Line 214, same comment. Instead, they did not detect a difference.

Line 219 – “the association was even larger” – there are post hoc statistical tests that can discern if the coefficients are truly statistically different from each other, which given substantial overlap in confidence intervals they may not be. Suggest revising.

Table 2 could also be formatted such that categories (e.g., age, race, are more explicitly grouped would improve readability). Bold text for statistically significant associations would be helpful. Similar feedback for Table 3.

Discussion:

Line 264 – although the coefficient is nominally larger, there are specific statistical tests that are used to determine whether the coefficients are statistically different – were these done?

Overall, the results are situated in the existing literature. The Discussion would be enhanced by some more mechanistic insights from the literature underlying the epidemiologic associations observed.

**********

6. PLOS authors have the option to publish the peer review history of their article (what does this mean?). If published, this will include your full peer review and any attached files.

If you choose “no”, your identity will remain anonymous but your review may still be made public.

**Do you want your identity to be public for this peer review?** For information about this choice, including consent withdrawal, please see our Privacy Policy.

Reviewer #1: No

Reviewer #2: No

Reviewer #3: No

Reviewer #4: No

**********

[NOTE: If reviewer comments were submitted as an attachment file, they will be attached to this email and accessible via the submission site. Please log into your account, locate the manuscript record, and check for the action link "View Attachments". If this link does not appear, there are no attachment files.]

While revising your submission, please upload your figure files to the Preflight Analysis and Conversion Engine (PACE) digital diagnostic tool, https://pacev2.apexcovantage.com/. PACE helps ensure that figures meet PLOS requirements. To use PACE, you must first register as a user. Registration is free. Then, login and navigate to the UPLOAD tab, where you will find detailed instructions on how to use the tool. If you encounter any issues or have any questions when using PACE, please email PLOS at figures@plos.org. Please note that Supporting Information files do not need this step.

# Author comment: Author response to Decision Letter 0

**DOI:** 10.1371/journal.pone.0309363.r002

27 Jul 2024

Please see document "Response to Reviewers" attached to submission. Pasted here without formatting:

Responses regarding journal requirements: (our responses are indicated by the bulleted arrow)

When submitting your revision, we need you to address these additional requirements.

1. Please ensure that your manuscript meets PLOS ONE's style requirements, including those for file naming. The PLOS ONE style templates can be found at

https://journals.plos.org/plosone/s/file?id=wjVg/PLOSOne_formatting_sample_main_body.pdf

and

https://journals.plos.org/plosone/s/file?id=ba62/PLOSOne_formatting_sample_title_authors_affiliations.pdf

� We ensured that our manuscript meets PLOS ONE’s style requirements.

2. Thank you for stating the following financial disclosure:

“Support for this research was provided by award UL1TR003017 from the National Center for Advancing Translational Sciences, a component of the National Institutes of Health and grant 74260 from the Robert Wood Johnson Foundation through its support of the Child Health Institute of New Jersey.”

Please state what role the funders took in the study. If the funders had no role, please state: "The funders had no role in study design, data collection and analysis, decision to publish, or preparation of the manuscript."

If this statement is not correct you must amend it as needed. Please include this amended Role of Funder statement in your cover letter; we will change the online submission form on your behalf.

� We added the suggested statement to the financial disclosure and included the revised financial disclosure in the cover letter, as directed.

3. We note that you have indicated that there are restrictions to data sharing for this study. PLOS only allows data to be available upon request if there are legal or ethical restrictions on sharing data publicly. For more information on unacceptable data access restrictions, please see http://journals.plos.org/plosone/s/data-availability#loc-unacceptable-data-access-restrictions.

Before we proceed with your manuscript, please address the following prompts:

a) If there are ethical or legal restrictions on sharing a de-identified data set, please explain them in detail (e.g., data contain potentially identifying or sensitive patient information, data are owned by a third-party organization, etc.) and who has imposed them (e.g., a Research Ethics Committee or Institutional Review Boar d, etc.). Please also provide contact information for a data access committee, ethics committee, or other institutional body to which data requests may be sent.

b) If there are no restrictions, please upload the minimal anonymized data set necessary to replicate your study findings to a stable, public repository and provide us with the relevant URLs, DOIs, or accession numbers. For a list of recommended repositories, please see

https://journals.plos.org/plosone/s/recommended-repositories. You also have the option of uploading the data as Supporting Information files, but we would recommend depositing data directly to a data repository if possible.

We will update your Data Availability statement on your behalf to reflect the information you provide.

[Note: HTML markup is below. Please do not edit.]

� We included the following data availability statement in our cover letter:

We used third-party data that are available to the public through a restricted data use contact from the Future of Families & Child Wellbeing Study at Princeton University. Details about the application process can be found at: https://ffcws.princeton.edu/restricted.

Responses to Reviewers

� We thank the reviewers for the very helpful comments. 

� Please note that in reanalyzing the data, we found that we were able to include a slightly larger number of cases in our analyses. This did not change any findings or interpretations. We also now focus exclusively on the measure of SGA that was based on LMP-based gestational age.

Reviewer #1: The article entitled “Small for gestational age and age at menarche in a contemporary population-based US sample” tested the hypothesis that SGA girls experience earlier menarche. Prior reports of racially homogenous populations have yielded mixed results. This analysis of a largely minority racial/ethnic population showed that maternal factors such as race/ethnicity and obesity influenced age of menarche, rather than SGA status. The data provides valuable information to add to our understanding of factors that influence pubertal onset. 

Specific comments:

1. Please provide more context to the FFCWB study, that may explain the demographic of the study population. 

� We now include the following paragraph on page 5, line 100:

The FFCWB cohort was population-based because it used a probability sample of cities, hospitals, and births within hospitals. Non-marital births were oversampled by design; that is, marital births were no longer included when hospital-specific quotas were reached. The reason for the oversampling was that unmarried parents and their children had been underrepresented in national U.S. datasets. About three-fourths of the mothers in the study were unmarried when they gave birth [25,26]. Because non-marital childbearing is strongly associated with poverty in the U.S., as is racial/ethnic minority status, the FFCWB sample had high fractions of poor, Black, and Hispanic parents.

2. The paragraph describing SGA (lines 113 to 125) was confusing. Line 114 – please clarify. Are the authors referring to determination of the gestational age? It’s also unclear why two different methods were needed to define SGA. subjects used the LMP vs the records-based measure. 

� We edited and clarified the paragraph describing SGA (page 6, line 116). We had been referring to the determination of gestational age, but that should be clear in the revised text, which now reads as follows:

SGA was defined as <10th percentile of sex-specific birth weight for gestational age. Gestational age was calculated as the number of completed weeks between the date of the first day of the mother’s last menstrual period (LMP) and the child’s birthday. The respondent’s birth weight for gestational age was compared to the national U.S. sex-specific reference distribution of LMP-based SGA from 1999–2000[28] when most of the FFCWB sample members were born. 

� Please note that we now focus exclusively on the measure of SGA that used LMP-based gestational age.

3. Lines 177/178: Is it the controls that were limited to births that were NOT low birthweight? 

� We clarified the text to indicate that we restricted the sample to children that weighed more than 2500 grams at birth in those analyses.

4. In Results, I recommend describing the difference in race/ethnicity between SGA vs not SGA. 

� Please see the following sentence on line 212 of the revised paper (bolding is for emphasis here):

Compared to girls who were not SGA, those who were SGA were more likely to have mothers who were non-Hispanic Black (60% vs. 48%), had Medicaid-financed births (76% vs. 63%), and smoked during pregnancy (44% vs. 21%), and were less likely to have mothers who were Hispanic (19% vs. 28%) and married (14% vs. 26%). 

4. Substantial discussion was given to the effect of obesity on early menarche but there was almost no discussion given for the effect of race/ethnicity on the same. Please elaborate on the current understanding of the relationship between race/ethnicity and pubertal timing (including if relevant, early childhood stress, environmental racism, Pediatrics (2022) 150 (2): e2021055595. https://doi.org/10.1542/peds.2021-055595). 

� We elaborated on the current understanding of the relationship between race/ethnicity and pubertal timing, bringing in the suggested article (line 288). We agree that this relationship was underdiscussed in the previous version of the paper.

Reviewer #2: The Authors hereby report the menarche onset in a pediatric cohort of small for gestational age females. The data are of interest and the cohort has a quite adequate dimension. There are two main concerns about this paper:

1. Table 1 should be moved into the Results sections, p column should be added in the right and not reported in legend 

� Done.

2. Why Authors chose 11 years old as a cut off for menarche? Precious menarche is considered as before 10 years-old, thus all considerations about whether SGA newborn females have precocious menarche more frequently rather than non-SGA should be done considering the 10 years-old cut-off.

� As we now indicate on line 51, no standard cut-off has been used in the literature; cut-offs have ranged from 10.5 to 12 and age 11 has been used in other studies (line 132). That said, we estimated supplementary models with age 10 as the cutoff (menarche before age 10), but with only 38 girls in our sample with early menarche defined this way, we were not able to estimate adjusted models. In the unadjusted models, SGA was not significantly associated with age in months at menarche or early menarche. We now point to not being able to use a more stringent cut-off as a limitation of our study (line 342). 

� Also, please note that we now focus exclusively on the measure of SGA that uses LMP-based gestational age.

Reviewer #3: Well written with statistics supporting the results. Also the arguments and rationale is well supported with references. Population sample is also presentation of contemporary US population. Overall good addition to the current literature.

� Thank you.

Reviewer #4: General comments: Sources of variation in age at menarche are a useful focus of investigation. The authors did not find associations between SGA and age at menarche. They did identify a number of other maternal factors related to age at menarche. The data are overall presented clearly, though there are some opportunities to improve formatting and readability suggested below, as well as questions regarding some of the statements (e.g., when the authors compare the value of two coefficients). 

1. Did the authors have any other health information about the girls at follow up (e.g., BMI) that also could yield more information, even if they did not have information about catch-up growth patterns? 

� We now report on supplemental results from models that interacted SGA and weight gain between birth and one year (line 262):

In supplementary models that interacted weight gain between birth and age 1 with SGA, we found no significant interactions. However, weight gain was associated with earlier menarche in those models (e.g., OLS coeff: -.75; CI: -1.36, -0.15) in the fully adjusted model for age at menarche in months and AOR: 1.13; CI: 1.02, 1.26 in the fully adjusted model for menarche before age 11) (not shown in tables). 

� Unfortunately, we do not have better health data to use for this supplementary analysis.

2. Sex differences are also interesting – can the authors also speculate as to the differences, if any, between maternal factors and pubertal outcomes in female infants as compared to male infants? 

� We now point to not being able to study linkages between SGA and puberty for males as a limitation of our study (line 344).

3. Abstract: The database consisting of mostly of non-marital births may not be representative of the U.S. population; the authors should comment in the Discussion on how their results may differ. That said, with this current focus, the database may teach us more about children deserving of additional study. 

� We now indicate in the abstract that the sample consisted of mostly non-marital births and highlight that it represents a policy-relevant population in the U.S. We elaborate on this point when commenting on the strengths and limitations of the study in the Discussion (line 326).

4. The authors describe “early menarche” as before age 11 years. Care should be taken to distinguish between “early menarche” (variation of typical) and “premature menarche” (implying the possibility of pathology). 

� We now make this distinction (line 53).

5. If the difference was not statistically significant, then the authors should not write “SGA was associated with slightly earlier menarche” which implies some meaningful difference. Instead, they can write, e.g., “We did not detect a difference.” 

� We changed the wording throughout to avoid such language.

6. The authors conclude that maternal non-Hispanic black race-ethnicity, Hispanic ethnicity, and pre-pregnancy obesity were associated with earlier age at menarche. If these factors related to SGA itself, could there be evidence of mediation? 

� This was an interesting comment. We ran supplementary models that did not include SGA to assess how much of a difference including SGA in the models affected the estimates for maternal non-Hispanic black race-ethnicity, Hispanic ethnicity, and pre-pregnancy obesity. We found that the estimates for all of these factors were very similar to those shown in the main models, suggesting no mediation. We could not find a straightforward way to motivate these supplementary analyses in the “supplementary analyses” section, so we ended up not reporting on these interesting supplementary results.

7. Early menarche is defined as before age 10.5y (line 47) but before age 11y in this manuscript; it is worth distinguishing the reasons. 

� As we now indicate on line 51, no standard cut-off has been used in the literature; cut-offs have ranged from 10.5 to 12 and age 11 has been used in other studies (line 132). That said, we estimated supplementary models with age 10 as the cutoff (menarche before age 10), but with only 38 girls in our sample with early menarche defined this way, we were not able to estimate adjusted models. In the unadjusted models, SGA was not significantly associated with age in months at menarche or early menarche. We now point to not being able to use a more stringent cut-off as a limitation of our study (line 342). 

8. Line 52 – The implied causal association between early menarche and mental health problems and antisocial behaviors is potentially fraught – adverse socioeconomic circumstances might explain both. The authors should either outline more about what is known about causality and/or make sure to differentiate. 

� We now make this important point as follows on line 56: 

Further, girls with early menarche are more likely to experience depressive symptoms and engage in health risk behaviors that adversely impact health in adulthood[7], although it has not been established whether those associations are confounded by socioeconomic factors. 

9. There is a fair number of missing measurements (859 out of a starting 2,295) – can the authors comment on any differences between those newborns with versus without available data (presumably maternal information at enrollment may be available)? 

� Done. This information is now presented in S1 Table.

10. What do the authors make of the finding that 10-11.6% of their sample was SGA (depending on measurement technique used)? This proportion is aligned with the expected proportion of <10% in the general population, even though their sample was designed to be enriched in individuals who might be expected to be at higher risk to have infants who are SGA? 

� We now focus exclusively on the analyses based on LMP-based gestational age. Using that measure, 11.7% of our sample was SGA and we highlight that this figure is expectedly higher than in the general population because of the largely disadvantaged population we studied (line 329). 

11. Line 152: BMI is weight in kg divided by height in meters (not centimeters) squared 

� We corrected this typographical error.

12. Present the differences between those included versus excluded from the sample in table form, i.e., include these details, since there were age and income differences.

� Done. We now present this information in S1 Table for all of the variables that are in our analyses.

13. Table 1 – should be presented in Results, and since this Table refers to the analysis cohort only, should be discussed after the in versus out of cohort.

� Done.

14. Age is reported in months to 2 significant digits. Suggest to 1 or no decimal points, and reporting 95% confidence intervals. See PMID 25877157. Suggest statistically significant d

Attachment Submitted filename: Response to Reviewers.docx

# Peer review: Decision Letter 1

**DOI:** 10.1371/journal.pone.0309363.r003

**Authors:** Inge Roggen

6 Aug 2024

PONE-D-24-13471R1Small for gestational age and age at menarche in a contemporary population-based U.S. samplePLOS ONE

Dear Dr. Sabu,

Thank you for submitting your manuscript to PLOS ONE. After careful consideration, we feel that it has merit but does not fully meet PLOS ONE’s publication criteria as it currently stands. Therefore, we invite you to submit a revised version of the manuscript that addresses the points raised during the review process. Please address these two minor (last) comments:1. Line 248/249: As there was no statistically significant difference in menarche age between SGA and non-SGA, the statement should be modified to state “There was no difference in early menarche between girls who were SGA vs. non-SGA.”

2. Please confirm in Table 2, for Adjusted maternal demographics, for Hispanic race ethnicity, if the upper CI is 3.61 or -3.61.

Please submit your revised manuscript by Sep 20 2024 11:59PM. If you will need more time than this to complete your revisions, please reply to this message or contact the journal office at plosone@plos.org. When you're ready to submit your revision, log on to https://www.editorialmanager.com/pone/ and select the 'Submissions Needing Revision' folder to locate your manuscript file.

Please include the following items when submitting your revised manuscript:

- A rebuttal letter that responds to each point raised by the academic editor and reviewer(s). You should upload this letter as a separate file labeled 'Response to Reviewers'.
- A marked-up copy of your manuscript that highlights changes made to the original version. You should upload this as a separate file labeled 'Revised Manuscript with Track Changes'.
- An unmarked version of your revised paper without tracked changes. You should upload this as a separate file labeled 'Manuscript'.

If you would like to make changes to your financial disclosure, please include your updated statement in your cover letter. Guidelines for resubmitting your figure files are available below the reviewer comments at the end of this letter.

If applicable, we recommend that you deposit your laboratory protocols in protocols.io to enhance the reproducibility of your results. Protocols.io assigns your protocol its own identifier (DOI) so that it can be cited independently in the future. For instructions see: https://journals.plos.org/plosone/s/submission-guidelines#loc-laboratory-protocols. Additionally, PLOS ONE offers an option for publishing peer-reviewed Lab Protocol articles, which describe protocols hosted on protocols.io. Read more information on sharing protocols at https://plos.org/protocols?utm_medium=editorial-email&utm_source=authorletters&utm_campaign=protocols.

We look forward to receiving your revised manuscript.

Kind regards,

Inge Roggen, M.D., Ph.D.

Academic Editor

PLOS ONE

Journal Requirements:

Please review your reference list to ensure that it is complete and correct. If you have cited papers that have been retracted, please include the rationale for doing so in the manuscript text, or remove these references and replace them with relevant current references. Any changes to the reference list should be mentioned in the rebuttal letter that accompanies your revised manuscript. If you need to cite a retracted article, indicate the article’s retracted status in the References list and also include a citation and full reference for the retraction notice.

[Note: HTML markup is below. Please do not edit.]

Reviewers' comments:

Reviewer's Responses to Questions

**Comments to the Author**

1. If the authors have adequately addressed your comments raised in a previous round of review and you feel that this manuscript is now acceptable for publication, you may indicate that here to bypass the “Comments to the Author” section, enter your conflict of interest statement in the “Confidential to Editor” section, and submit your "Accept" recommendation.

Reviewer #1: All comments have been addressed

Reviewer #3: All comments have been addressed

Reviewer #4: All comments have been addressed

**********

2. Is the manuscript technically sound, and do the data support the conclusions?

The manuscript must describe a technically sound piece of scientific research with data that supports the conclusions. Experiments must have been conducted rigorously, with appropriate controls, replication, and sample sizes. The conclusions must be drawn appropriately based on the data presented. 

Reviewer #1: Partly

Reviewer #3: Yes

Reviewer #4: Yes

**********

3. Has the statistical analysis been performed appropriately and rigorously? 

Reviewer #1: Yes

Reviewer #3: Yes

Reviewer #4: Yes

**********

4. Have the authors made all data underlying the findings in their manuscript fully available?

The PLOS Data policy requires authors to make all data underlying the findings described in their manuscript fully available without restriction, with rare exception (please refer to the Data Availability Statement in the manuscript PDF file). The data should be provided as part of the manuscript or its supporting information, or deposited to a public repository. For example, in addition to summary statistics, the data points behind means, medians and variance measures should be available. If there are restrictions on publicly sharing data—e.g. participant privacy or use of data from a third party—those must be specified.

Reviewer #1: Yes

Reviewer #3: Yes

Reviewer #4: Yes

**********

5. Is the manuscript presented in an intelligible fashion and written in standard English?

PLOS ONE does not copyedit accepted manuscripts, so the language in submitted articles must be clear, correct, and unambiguous. Any typographical or grammatical errors should be corrected at revision, so please note any specific errors here.

Reviewer #1: Yes

Reviewer #3: Yes

Reviewer #4: Yes

**********

6. Review Comments to the Author

Please use the space provided to explain your answers to the questions above. You may also include additional comments for the author, including concerns about dual publication, research ethics, or publication ethics. (Please upload your review as an attachment if it exceeds 20,000 characters)

Reviewer #1: The responses to prior comments have been adequately addressed. I have two minor comments:

1. Line 248/249: As there was no statistically significant difference in menarche age between SGA and non-SGA, the statement should be modified to state “There was no difference in early menarche between girls who were SGA vs. non-SGA.”

2. Please confirm in Table 2, for Adjusted maternal demographics, for Hispanic race ethnicity, if the upper CI is 3.61 or -3.61.

Reviewer #3: Author has responded to all the comments and the study add good contribution to the literature in maternal and child health domain.

Reviewer #4: From above: If the authors have adequately addressed your comments raised in a previous round of review and you feel that this manuscript is now acceptable for publication, you may indicate that here to bypass the “Comments to the Author” section, enter your conflict of interest statement in the “Confidential to Editor” section, and submit your "Accept" recommendation.

**********

7. PLOS authors have the option to publish the peer review history of their article (what does this mean?). If published, this will include your full peer review and any attached files.

If you choose “no”, your identity will remain anonymous but your review may still be made public.

**Do you want your identity to be public for this peer review?** For information about this choice, including consent withdrawal, please see our Privacy Policy.

Reviewer #1: No

Reviewer #3: **Yes: **Rabia Baloch

Reviewer #4: No

**********

[NOTE: If reviewer comments were submitted as an attachment file, they will be attached to this email and accessible via the submission site. Please log into your account, locate the manuscript record, and check for the action link "View Attachments". If this link does not appear, there are no attachment files.]

While revising your submission, please upload your figure files to the Preflight Analysis and Conversion Engine (PACE) digital diagnostic tool, https://pacev2.apexcovantage.com/. PACE helps ensure that figures meet PLOS requirements. To use PACE, you must first register as a user. Registration is free. Then, login and navigate to the UPLOAD tab, where you will find detailed instructions on how to use the tool. If you encounter any issues or have any questions when using PACE, please email PLOS at figures@plos.org. Please note that Supporting Information files do not need this step.

# Author comment: Author response to Decision Letter 1

**DOI:** 10.1371/journal.pone.0309363.r004

8 Aug 2024

Pasted without formatting: 

1. Line 248/249: As there was no statistically significant difference in menarche age between SGA and non-SGA, the statement should be modified to state “There was no difference in early menarche between girls who were SGA vs. non-SGA.”

� The line number listed by the reviewer did not correlate to a relevant sentence. But we noted a similar statement earlier in our paper. We made this change (line 197-198) that now reads: 

There was no difference in age at menarche between girls who were born SGA vs. those who were not born SGA.

2. Please confirm in Table 2, for Adjusted maternal demographics, for Hispanic race ethnicity, if the upper CI is 3.61 or -3.61.

� Thank you for catching this typo. The upper CI should be -3.61. We made this change.

Attachment Submitted filename: Response to Reviewers.docx

# Editor report: Decision Letter 2

**DOI:** 10.1371/journal.pone.0309363.r005

**Authors:** Inge Roggen

12 Aug 2024

Small for gestational age and age at menarche in a contemporary population-based U.S. sample

PONE-D-24-13471R2

Dear Dr. Sabu,

We’re pleased to inform you that your manuscript has been judged scientifically suitable for publication and will be formally accepted for publication once it meets all outstanding technical requirements.

Within one week, you’ll receive an e-mail detailing the required amendments. When these have been addressed, you’ll receive a formal acceptance letter and your manuscript will be scheduled for publication.

An invoice will be generated when your article is formally accepted. Please note, if your institution has a publishing partnership with PLOS and your article meets the relevant criteria, all or part of your publication costs will be covered. Please make sure your user information is up-to-date by logging into Editorial Manager at Editorial Manager® and clicking the ‘Update My Information' link at the top of the page. If you have any questions relating to publication charges, please contact our Author Billing department directly at authorbilling@plos.org.

If your institution or institutions have a press office, please notify them about your upcoming paper to help maximize its impact. If they’ll be preparing press materials, please inform our press team as soon as possible -- no later than 48 hours after receiving the formal acceptance. Your manuscript will remain under strict press embargo until 2 pm Eastern Time on the date of publication. For more information, please contact onepress@plos.org.

Kind regards,

Inge Roggen, M.D., Ph.D.

Academic Editor

PLOS ONE

Additional Editor Comments (optional):

Reviewers' comments:

# Editor report: Acceptance letter

**DOI:** 10.1371/journal.pone.0309363.r006

**Authors:** Inge Roggen

27 Aug 2024

PONE-D-24-13471R2 

PLOS ONE

Dear Dr. Sabu, 

I'm pleased to inform you that your manuscript has been deemed suitable for publication in PLOS ONE. Congratulations! Your manuscript is now being handed over to our production team.

At this stage, our production department will prepare your paper for publication. This includes ensuring the following:

* All references, tables, and figures are properly cited

* All relevant supporting information is included in the manuscript submission,

* There are no issues that prevent the paper from being properly typeset

If revisions are needed, the production department will contact you directly to resolve them. If no revisions are needed, you will receive an email when the publication date has been set. At this time, we do not offer pre-publication proofs to authors during production of the accepted work. Please keep in mind that we are working through a large volume of accepted articles, so please give us a few weeks to review your paper and let you know the next and final steps. 

Lastly, if your institution or institutions have a press office, please let them know about your upcoming paper now to help maximize its impact. If they'll be preparing press materials, please inform our press team within the next 48 hours. Your manuscript will remain under strict press embargo until 2 pm Eastern Time on the date of publication. For more information, please contact onepress@plos.org.

If we can help with anything else, please email us at customercare@plos.org.

Thank you for submitting your work to PLOS ONE and supporting open access. 

Kind regards, 

PLOS ONE Editorial Office Staff

on behalf of

Prof. Inge Roggen 

Academic Editor

PLOS ONE
